# Myocarditis as a lupus challenge: two case reports

**DOI:** 10.1186/s13256-019-2242-1

**Published:** 2019-11-20

**Authors:** Shamma Ahmad Al-Nokhatha, Hiba Ibrahim Khogali, Maryam Abdulla Al Shehhi, Imad Tarik Jassim

**Affiliations:** Department of Rheumatology, Internal Medicine, Tawam Hospital, Al Ain, United Arab Emirates

**Keywords:** Systemic lupus erythematosus, Mixed connective tissue disease, Myocarditis, Mycophenolate mofetil, Heart failure

## Abstract

**Background:**

Myocarditis is an uncommon manifestation of systemic lupus erythematosus in which the clinical presentation can range from subclinical to life-threatening. We report cases of two patients who presented to our hospital with myocarditis as an initial manifestation of systemic lupus erythematosus despite negative results of extensive workup that excluded other diagnoses. The mainstays of treatment are corticosteroids, immunosuppressive agents, and anti-heart failure medications, with use of the latter being case-specific. Mycophenolate mofetil was the cornerstone of the proposed treatment for induction of remission, although it is well known to be used as a maintenance therapy in lupus myocarditis.

**Case presentation:**

Both Emirati patients described satisfied the diagnostic criteria for mixed connective tissue disease (systemic lupus predominant) and systemic lupus erythematous. Other differential diagnoses of myocarditis were excluded. The patients were started on pulsed steroid followed by oral steroid, with hydroxychloroquine, mycophenolate mofetil, and anti-heart failure medications used as needed. Dramatic responses were noted in the first few weeks in terms of symptoms.

**Conclusion:**

Early recognition and treatment of lupus myocarditis is needed to avoid fatal consequences.

## Introduction

Systemic lupus erythematosus (SLE) is a multisystem autoimmune disease with cardiac involvement in up to 50% of cases [1]. It can manifest as pericardial disease, myocardial dysfunction, valvular heart disease, conduction system abnormalities, or atheromatous disease. Myocarditis is one form of cardiac involvement in SLE; it is reported to occur in 5–10% of symptomatic patients, whereas around 50% have a subclinical form proved by autopsy. The clinical presentation of myocarditis in SLE ranges from asymptomatic patients with self-limited disease to fulminant heart failure that can lead to death. The management of lupus myocarditis is challenging, and few studies have described the optimal treatment options [2–4]. In the present report, to shed light on this condition and the importance of mycophenolate mofetil (MMF) in therapy induction, we describe two patients with such a presentation. MMF was the cornerstone of the proposed treatment for induction of remission, although it is well known to be used as a maintenance therapy in lupus myocarditis.

## Case presentations

### Patient 1

Patient 1 was a 41-year-old Emirati woman who was a housewife with known hypertension, hypothyroidism, and asthma. She presented to our hospital with a 1-month history of fever associated with chills, rigors, pleuritic chest pain, pain in the small joints of the hand, cold in the extremities, and photosensitivity. She was also noted to have a 1-year history of progressive fatigue, arthralgia, 20-kg weight loss, and intermittent low- and high-grade fever. On examination, she was febrile with a temperature of 100.76 °F (38.2 °C) and a heart rate of 105 beats/minute. Her vital signs were otherwise unremarkable. Generally, she looked pale. Head examination revealed a diffuse alopecia with no oral ulcers. Cardiac examination showed normal heart sounds without murmur. Pulmonary examination revealed normal air entry bilaterally with no added sounds. The patient had palpable cervical lymphadenopathy and no rashes. Musculoskeletal examination showed sclerodactyly, hand edema with no joint tenderness, and preserved range of motion. The patient had no Raynaud’s phenomenon, splinter hemorrhages, or telangiectasia. The remainder of her examination was unremarkable. She had no relevant social or family history.

Laboratory investigations revealed pancytopenia with a white blood cell count of 1.4 × 10^9^ cells/L, platelet count of 98 × 10^9^/L, hemoglobin 7.8 gm/dl with low mean corpuscular volume and hematocrit, a negative Coombs test result, and high inflammatory markers (erythrocyte sedimentation rate [ESR], 80 mm/hour [normal range, 0–20]; C-reactive protein [CRP], 180 mg/L [normal range, 0–8]; and ferritin, 7654 μg/L). The patient had a normal renal function test result and mild derangement in liver function enzymes. She had mildly raised creatine kinase MB (CK-MB) at 13.8 ng/ml (normal range, 0.6–6.3 ng/ml) and troponin at 0.15 ng/ml (normal, <0.1 ng/ml), and she had a normal total CK concentration. The results of an extensive infectious screen were negative. Immunological tests showed a high positive antinuclear antibodies (ANA) titer of 1:2560 with speckled pattern, negative anti–double-stranded DNA antibodies, negative antiphospholipid antibodies, and low complement levels. Her antiribonucleoprotein antibodies, anti-Sm, and anti-Ro antibodies were positive.

A chest radiograph showed cardiomegaly without effusion or infiltrates. Abdominal ultrasound showed no intra- or extrahepatic dilation, with normal common bile duct (CBD). Computed tomography (CT) of the chest suggested mild basal lung fibrosis. Apart from T-wave inversion on anterolateral chest leads, the result of the patient’s electrocardiography (ECG) was unremarkable. Echocardiography (ECHO) revealed moderate regional wall systolic dysfunction (ejection Fraction [EF], 40%) with moderate pulmonary hypertension. The result of bone marrow biopsy was consistent with normochromic anemia only.

### Patient 2

Patient 2 was a previously healthy 33-year-old single Emirati woman who was referred to our hospital with suspicion of SLE for further investigations. Three months prior to presentation, she had complained of profound fatigue, widespread arthralgia, ongoing dyspnea, and chest tightness. On examination, the patient was afebrile with normal vital parameters. She appeared weak and pale. No malar rash was noted. Livedo reticularis was present on her chest and back. Cardiac examination revealed normal heart sounds without murmur. Pulmonary examination revealed bilateral decreased breath sounds with bilateral basal crackles. Lower limb edema was also noted. The remainder of her examination was unremarkable. She had no relevant social or family history.

Laboratory investigations revealed normal complete blood count and renal and liver function test results, raised inflammatory markers (ESR, 103 mm/hour; CRP, 90 mg/L), a normal cardiac profile (CKMB, 1.9 ng/ml [normal range, 0.6–6.3 ng/ml]; troponin 0.03 ng/ml [normal, < 0.1 ng/ml]), and a normal total CK level.

Immunological workup showed a high positive ANA titer (1:2560) with speckled pattern, positive anti–double-stranded DNA antibodies, negative antiphospholipid antibodies, normal complements, and positive anti-Ro/La antibodies.

**Fig. 1:**
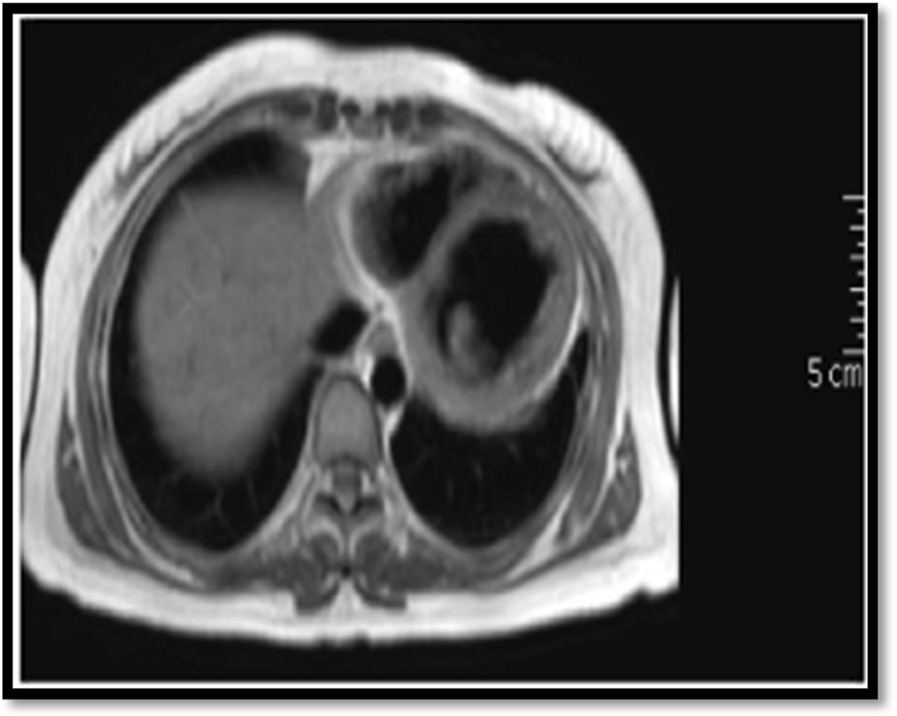
Cardiac magnetic resonance imaging showing subepicardial late gadolinium enhancement of the basal inferior, anteroseptal, and inferoseptal segments. The findings indicate dilated cardiomyopathy with previous myocarditis (not active anymore, indicated by lack of myocardial edema)

Chest radiography showed cardiomegaly. CT of the chest showed pleural scarring with minimal left-sided pleural effusion in keeping with pleuritis, and ECG showed normal sinus rhythm. ECHO showed severely depressed left ventricular systolic function (EF, 35%) with traces of pericardial effusion. Cardiac magnetic resonance imaging confirmed the diagnosis of myocarditis (Fig. 1). Findings of CT of the coronary arteries were normal.

### Management

Our two patients satisfied the criteria for mixed connective tissue disease (systemic lupus predominant) and SLE, respectively. Both of them were started on a pulsed dose of corticosteroid (500 mg intravenously for 3 days) followed by oral steroid. Full doses of hydroxychloroquine, MMF, and anti-heart failure medications as needed were also prescribed. Dramatic responses were noted in the first few weeks in terms of symptoms. The EF improved to 60% in patient 1, whereas it remained the same in patient 2 after repeating the ECHO within 1 year.

## Discussion

We report two patients diagnosed with lupus myocarditis who had clinical improvement after starting immunosuppression. To our knowledge, the present report is one of few to demonstrate the importance of MMF in particular for remission induction. There are no published guidelines on the diagnosis of lupus myocarditis. Owing to the limited tools for detecting subclinical myocarditis, more and more cases go undetected and thereafter not managed. Current treatment strategies are based on general consensus and clinical experiences rather than randomized controlled trials.

Endomyocardial biopsy is the gold standard in diagnosing myocarditis. It is an invasive measure with a risk-related procedure and limited availability in clinical practice. Its diagnostic yield is low at only 10–20%. It can exclude other diagnoses. Therefore, myocarditis diagnosis in SLE still depends largely on clinical suspicion and ECHO findings [5].

There are few case reports of acute myocarditis and heart failure as an initial SLE presentation. The therapeutic approach for myocarditis starts from standard measures with supportive care as first-line therapy, whereas anti-heart failure medications and additional treatment are prescribed on the basis of underlying etiology [6]. Few published data in the literature highlighted the optimal immunosuppressive medications following corticosteroid use as the mainstay of treatment for myocarditis in patients with SLE. These medications include cyclophosphamide, immunoglobulin, and plasma exchange. However, few case reports have described the use of MMF for induction of remission, although it is well known to be used as a maintenance therapy. A retrospective, multicenter study from three French university hospitals reported that 2 of 29 cases were treated with MMF as first-line immunosuppressive therapy in lupus myocarditis. The median follow-up was 37 months, and significant improvements were noted in left ventricular EF and overall cardiac recovery in patients not treated with cyclophosphamide (MMF, intravenous immunoglobulin, and plasma exchange) [1, 7]. A recent case series of eight patients describing the use of ^18^F-fluorodeoxyglucose–positron emission tomography (^18^F-FDG-PET)/CT in diagnosing lupus myocarditis, seven of eight patients were treated with MMF with a goal dose of 3 g/day in divided doses following steroids. Of these seven patients, two were followed with ^18^F-FDG-PET/CT: One patient had no myocardial uptake after 5 months, and the other one had a decrease in FDG uptake measured by a standardized uptake value factor of 3 at 13 months. Transthoracic echocardiography showed an EF of 57% (range, 50–60%) with normal wall motion in another four patients within 6–8 months. The last patient was lost to follow-up [8]. It seems to be a reasonable choice to consider MMF as a suitable induction therapy for lupus myocarditis. There are no placebo-controlled, double-blind, randomized, controlled trials specifically designed to assess the use of MMF in nonrenal lupus and myocarditis in particular [9].

Further efforts to collect relevant data for those patients should include a multicenter registry in order to establish well-designed criteria and guidelines for an optimal treatment regimen.

## Conclusion

Myocarditis is one of the most challenging diagnoses; early recognition and treatment with aggressive immunosuppressive therapy can result in a good clinical outcome in most cases. Lupus myocarditis treatment is still not well established. MMF can be helpful in induction of remission, as we have highlighted in reporting these two cases.

## Data Availability

All data generated or analyzed during this study are included in this published article.

## Abbreviations

ANA: Antinuclear antibodies
CRP: C-reactive protein
CK: Creatine kinase
CT: Computed tomography
ECG: Electrocardiography
ECHO: Echocardiography
EF: Ejection fraction
ESR: Erythrocyte sedimentation rate
^18^F-FDG-PET: ^18^F-fluorodeoxyglucose–positron emission tomography
MMF: Mycophenolate mofetil
SLE: Systemic lupus erythematosus
